# Resistome and Genome Analysis of an Extensively Drug-Resistant *Klebsiella michiganensis* KMIB106: Characterization of a Novel KPC Plasmid pB106-1 and a Novel Cointegrate Plasmid pB106-IMP Harboring *bla*_IMP-4_ and *bla*_SHV-12_

**DOI:** 10.3390/antibiotics12091463

**Published:** 2023-09-20

**Authors:** Linjing Wang, Haijun Chen, Wanting Liu, Ling Yang, Zhenbo Xu, Dingqiang Chen

**Affiliations:** 1 Microbiome Medicine Center, Department of Laboratory Medicine, Zhujiang Hospital, Southern Medical University, Guangzhou 510280, China; 2 Department of Laboratory Medicine, The First Affiliated Hospital of Guangzhou Medical University, Guangzhou 510120, China; 3 School of Food Science and Engineering, Research Institute for Food Nutrition and Human Health, Guangdong Province Key Laboratory for Green Processing of Natural Products and Product Safety, Engineering Research Center of Starch and Vegetable Protein Processing Ministry of Education, South China University of Technology, Guangzhou 510640, China; 4 Department of Laboratory Medicine, The Second Affiliated Hospital of Shantou University Medical College, Shantou 515141, China

**Keywords:** *Klebsiella michiganensis*, *bla*_IMP-4_, *bla*_SHV-12_, IS*26*, cointegrate plasmid

## Abstract

*Klebsiella michiganensis* is a recently emerging human pathogen causing nosocomial infections. This study aimed to characterize the complete genome sequence of a clinical *Klebsiella michiganensis* strain KMIB106 which exhibited extensive drug-resistance. The whole genome of the strain was sequenced using PacBio RS III systems and Illumina Nextseq 500. Annotation, transposable elements and resistance gene identification were analyzed by RAST, prokka and Plasmid Finder, respectively. According to the results, KMIB106 was resistant to multiple antimicrobials, including carbapenems, but it remained susceptible to aztreonam. The genome of KMIB106 consisted of a single chromosome and three predicted plasmids. Importantly, a novel KPC plasmid pB106-1 was found to carry the array of resistance genes in a highly different order in its variable regions, including *mphA*, *msrE*, *mphE*, *ARR-3*, *addA16*, *sul1*, *dfrA27*, *tetD* and *fosA3*. Plasmid pB106-2 is a typical IncFII plasmid with no resistant gene. Plasmid pB106-IMP consists of the IncN and IncX3 backbones, and two resistance genes, *bla*_IMP-4_ and *bla*_SHV-12_, were identified. Our study for the first time reported an extensively drug-resistant *Klebsiella michiganensis* strain recovered from a child with a respiratory infection in Southern China, which carries three mega plasmids, with pB106-1 firstly identified to carry an array of resistance genes in a distinctive order, and pB106-IMP identified as a novel IncN-IncX3 cointegrate plasmid harboring two resistance genes *bla*_IMP-4_ and *bla*_SHV-12_.

## 1. Introduction

*Klebsiella michiganensis* was first identified in Michigan from a toothbrush holder in 2012 and was initially identified as *Klebsiella oxytopca* due to their highly similar *16S rRNA* sequences (approximately 99% identity) until it was then recognized as a new species by DNA-DNA hybridization. Since then, *K. michiganensis* has been recognized as a potential emerging human pathogen responsible for nosocomial infections. Commonly isolated from blood and rectal swabs, this bacterium often infects immune compromised patients, with associated infection cases being increasingly reported in recent years. In addition, the antimicrobial resistance in *K. michiganensis* has become a major concern in the therapeutic treatment of relevant infections, especially for *K. michiganensis* exhibiting multidrug resistance (MDR) [1,2,3]. Also, the carriage of various antibiotic resistance genes has been found to be associated with the multidrug resistance phenotype [1,2,3]. In particular, over the past decades, carbapenemase-producing *Klebsiella* spp. and their carriage of various carbapenem resistance genes have been frequently identified, which has become a leading challenge to clinicians [2,4]. For *K. michiganensis*, strains harboring two to three carbapenem resistance genes have been previously reported in South Africa and China [5,6].

Carbapenemase is able to hydrolyze most of the β-lactam antibiotics [7]. As an Ambler class B metallo-β-lactamase, imipenemase (IMP) confers resistance to all the β-lactam antibiotics except for the monocyclic β-lactam antibiotic and is not susceptible to β-lactamases inhibitors [8]. Up to now, a total of 62 IMP variants have been identified (https://bitbucket.org/genomicepidemiology/resfinder_db/src/master/beta-lactam.fsa; accessed on 20 October 2020). Among them, IMP-4 is the prevalent imipenemase in China [9], which was first detected in *Acinetobacter* spp. in Hong Kong in 2001 and ever since has been spread worldwide [10]. The carriage of the *bla_IMP-4_* gene provides the host strain with high minimum inhibitory concentration (MIC) values for almost all β-lactams. Wide dissemination of IMP is often due to various transferable plasmids [11,12,13,14], and IMP genes are frequently reported to be carried in combination with other resistance genes. As a consequence, the coexistence of IMP and other resistance genes in a plasmid confers higher resistance to antibiotics, raising a significant challenge for clinical treatment [15].

Plasmids are DNA genetic elements in bacteria that can replicate themselves. Typical plasmids are composed of a backbone and several variable regions, which usually carries different species and different numbers of mobilizable elements including transposons, integrons and insertion sequences (ISs). Moreover, these mobilizable elements usually carry antibiotic resistance genes; therefore, horizontal gene transfer mediated by plasmids makes a significant contribution to the dissemination of antibiotic resistance genes between different bacterial species [16].

In this study, an extensively drug-resistant *K. michiganensis* strain recovered from a child with a respiratory infection from a teaching hospital in Southern China was subjected to genomic sequencing and further bioinformative analysis. For the first time, we have identified three mega plasmids, with pB106-1 first identified to carry an array of resistance genes in a distinctive order which provides important evidence on the evolution of antimicrobial resistance in *K. michiganensis*

## 2. Materials and Methods

### 2.1. Isolation of the K. michiganensis KMIB106 Strain

The *K. michiganensis* strain KMIB106 was isolated from a 7-year-old girl in the First Affiliated Hospital of Guangzhou Medical University (FAHGMU) in Southern China. The child was hospitalized with a diagnosis of severe pneumonia in April 2019, who then suffered from fever with a maximum temperature of 41 °C and paroxysmal continuous cough. During the treatment, 3 different types of bacterial strains were isolated from independent sputum samples, including *Acinetobacter baumannii*, *K. michiganensis* and *Elizabethkingia meningoseptica*.

### 2.2. Bacterial Identification and Antimicrobial Susceptibility Testing

For all bacterial strains, biochemical identification to species level and the susceptibility test of 20 common antibiotics were performed using the Vitek2^TM^ Automated Susceptibility System [17], including ceftazidime, cefepime, cefuroxime sodium, ceftriaxone, cefuroxime axetil, cefoxitin, piperacillin/tazobactam, amoxicillin/clavulanic acid, ticacillin/clavulanic acid, compound sulfamethoxazole, minocycline, doxycycline, imipenem, ertapenem, colistin, aztreonam, amikacin, tobramycin, ciprofloxacin and levofloxacin. The MICs were interpreted according to the guidelines recommended by the Clinical and Laboratory Standards Institute (CLSI).

### 2.3. Genome Sequencing and De Novo Assembly and Correction

Bacterial genome sequencing was performed by PacBio RS III systems and Illumina Nextseq 500. For sequencing by PacBio RS III, genomic DNA was extracted using the CTAB method, followed by a concentration and quality test. Then, genomic DNA was fragmented with G-tubes and end-repaired to prepare SMRTbell DNA template libraries (with fragment size of >10 Kb selected by the Blue Pippin system). After the average fragment size was estimated on a Bioanalyzer 2100, SMRT sequencing was performed on the Pacific Biosciences Sequel. For sequencing by Illumina Nextseq 500, qualified genomic DNA was sonicated randomly, then end-repaired, A-tailed, and adaptor-ligated using NEBNext^®^ ΜLtra™ DNA Library Prep Kit for Illumina (NEB, TX, USA). DNA fragments with a length of 300–400 bp were enriched by PCR. PCR products were purified using the AMPure XP system (Beckman Coulter, Brea, CA, USA). The libraries were analyzed for size distribution by a 2100 Bioanalyzer (Agilent, Santa Clara, CA, USA) and quantified using real-time PCR. Genome sequencing was performed on the Illumina Novaseq 6000 sequencer using the pair-end technology. Continuous long reads obtained from SMRT sequencing runs were used for further de novo assembly using Falcon (version 0.3.0) [18]. Continuous long reads obtained from ONT sequencing were used for de novo assembly using Flye (version 2.8.1-b1676) [19]. Raw data from the Illumina platform were filtered using FASTP (version 0.20.0) [20] by the following standards: (1) removing reads with ≥10% unidentified nucleotides (N); (2) removing reads with ≥50% bases having Phred quality scores ≤ 20; (3) removing reads aligned to the barcode adapter. After filtering, the clean reads were used to correct the genome sequences to improve the quality of the assembly and determine the final genome sequences using Pilon (version 1.23) [21].

### 2.4. Genome Annotation and Analysis

The ORFs (open reading frames) were predicted using the NCBI prokaryotic genome annotation pipeline. For noncoding RNAs, rRNAs, tRNAs and sRNAs, predictions were carried out using rRNAmmer (version 1.2), tRNAscan (version 1.3.1) and cmscan (version 1.1.2), respectively. Gene islands and transposons were predicted using IslandPath-DIMOB (version 1.0.0) and Transposon PSI (version 20100822). Interspersed repeat elements were identified by Repeat Masker (version 4.0.5). The acquired genome sequences were further annotated with RAST and prokka tools [22,23]. The replicon types of plasmids were identified with Plasmid Finder-1.3 [24] and the resistance genes of plasmids were also predicted with ResFinder [25]. The genome of KMIB106 was searched for in CRISPR regions over the public databases using CRISPRfinder [26].

### 2.5. GenBank Accession Number

The whole genome sequences of KMIB106, including the chromosome and 3 plasmids, pB106-1, pB106-2 and pB106-IMP, were submitted to GenBank nucleotide sequence database under accession numbers CP067093.1, CP067094.1, CP067095.1 and CP067096.1, respectively.

## 3. Results

### 3.1. Antibiotic Susceptibilities

The antimicrobial susceptibility testing of the *K. michiganensis* strain KMIB106 was performed on 20 antibiotics, with MICs interpretation shown in Table 1. According to the results, the *K. michiganensis* strain KMIB106 exhibited an extensive drug-resistance, with resistance to 14 different compounds, including ceftazidime (MIC ≥ 64), cefepime (MIC ≥ 32), cefuroxime sodium (MIC ≥ 64), ceftriaxone (MIC ≥ 64), cefuroxime axetil (MIC ≥ 64), cefoxitin (MIC ≥ 64), piperacillin/tazobactam (MIC ≥ 128), amoxicillin/clavulanic acid (MIC ≥ 32), ticacillin/clavulanic acid (MIC ≥ 128), compound sulfamethoxazole (MIC ≥ 320/80), minocycline (MIC ≥ 16), doxycycline (MIC ≥ 16), imipenem (MIC ≥ 16) and ertapenem (MIC ≥ 8). In contrast, the *K. michiganensis* strain KMIB106 was susceptible to tigecycline (MIC ≤ 0.5), colistin (MIC ≤ 0.5), aztreonam (MIC ≤ 1), amikacin (MIC ≤ 2), tobramycin (MIC ≤ 1), ciprofloxacin (MIC ≤ 0.25) and levofloxacin (MIC ≤ 0.12).

### 3.2. Genomic Analysis of Bacterial Chromosome

The average genome sequencing depth was more than 100X in coverage, including 1.2 G and 1.7 G data acquired from PacBio and Illumina platforms, respectively. The genome of the *K. michiganensis* strain KMIB106 was assembled into a single linear chromosome and three circular plasmids (Figure S1). The chromosome consisted of 5,907,184 bp with an overall G+C content of 55.48%. Among the 5667 predicted CDs from the chromosome, a total of 86 tRNA and 25 rRNA were identified, with 2 CRISPR-cas sequences further identified by the prokka and the CRISPRfinder server.

### 3.3. Profile of Resistance Gene

Analyses of antimicrobial resistance genes were performed on the genome of the *K. michiganensis* strain KMIB106 via ResFinder. A fluoroquinolone resistance gene *oqxB* [27] and a beta-lactam resistance gene *bla*_OXY-4-1_ were found in the chromosome (Table 2). Nine resistance genes were found in the novel KPC plasmid pB106-1, including genes conferring resistance to macrolide (*mphA*, *msrE*, *mphE*), rifamycin (*ARR-3*), aminoglycosides (*addA16*), folate pathway antagonist (*sul1*, *dfrA27*), fosfomycin (*fosA3*) and tetracycline (*tetD*). In addition, 2 β-lactamase genes *bla*_SHV-12_ and *bla*_IMP-4_ were found in plasmid pB106-IMP.

### 3.4. Correlation between Antimicrobial Resistance Phenotype and Genes Profile

The correlation between antimicrobial resistance phenotype and resistance genes profile was further analyzed in the *K. michiganensis* strain KMIB106. As shown in Table 2, the antimicrobial resistance phenotype was in accordance with the carriage of relevant resistance gene. For each antimicrobial agent group to which the *K. michiganensis* strain KMIB106 was resistant, the strain carried the relevant resistance gene. Remarkably, high MIC values were obtained for Beta-lactams and compound sulfamethoxazole, for which KMIB106 harbors 3 *bla* genes and *sul1* with *dfrA27*, respectively.

### 3.5. Genomic Analysis of the Novel KPC Plasmid pB106-1

**Figure 1:**
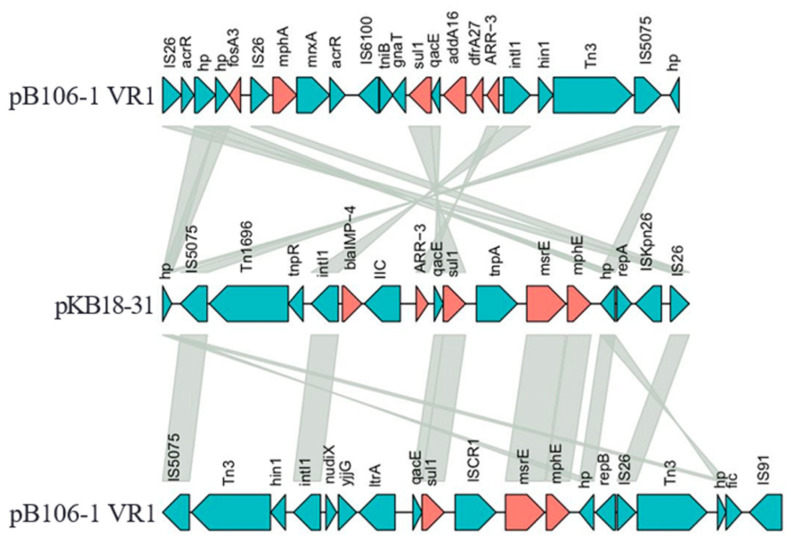
The comparison between variable regions of plasmid pB106-1 and plasmid pKB18-31; genes are denoted by arrows, resistance genes are highlighted in red, other genes are shown in green and the gray area represents that the similarity between these sequences is more than 99%.

The size of plasmid pB106-1 was 284,262 bp, with the average G+C content at 47.04% and the total number of predicted CDs at 382. The sequence of plasmid pB106-1 showed high similarity (91% coverage, 99% identity) with plasmid pKP18-31-IMP-KPC (accession number: MN661402.1) (Figure 1). Both plasmid pB106-1 and pKP18-31-IMP-KPC contained a highly similar 230 kb backbone with a slight difference in the variable regions. In plasmid pB106-1, this backbone shared 80% identity with 41 plasmids in GenBank, with more than 95% query coverage. Among such 41 plasmids, 20, 7 and 4 were from *K. pneumoniae*, *K. michiganensis* and *Raoultella planticola*, suggesting the plasmid pB106-1 was likely derived from *K. pneumoniae*. This backbone of plasmid pB106-1 contained sequences responsible for plasmid maintenance (*stbE* and *stbD*) and conjugative transfer (*traD*, *trbB*, *traE*, *trbN* and *trhU*). Also, the replication sequences could be incorporated into two replicons (*repB* and *repHI5B*). Analyzed by ResFinder, plasmid pB106-1 did not belong to any incompatibility groups of Enterobacteriaceae.

Importantly, in plasmid pB106-1, the 18,748 bp variable region VR1 (nt 78,572 to 97,319) contained three fragments, including a composite transposon organized as *IS26-acrR-hp-hp-fosA3-IS26-mphA-mrxA-acrR-IS600*, a class 1 integron and a unique transposon Tn3 (lack of resolvase tnpR). Most of the resistance genes in plasmid pB106-1 were located in the VR1 to form a related subsidiary drug-resistant area. The VR1 was inserted in the same position in plasmid pKP18-31-IMP, KPC. However, the array of genes was arranged in a different order from pB106-1 and pKP18-31-IMP, KPC. These two variable regions both contained IS*26* and IS*5075* as boundaries and their class 1 integron had the same 5′-conserved segment (5′-CS), 3′-CS and *ARR-3*, whereas the variable region of plasmid pKP18-31-IMP, KPC contained three different fragments, a unit transposon Tn1696, a class 1 integron and a composite transposon organized as *ISCR1-msrE-mphE-hp-repA-ISKpn26-IS26* (Figure 1).

In plasmid pB106-1, another 23,276 bp variable region VR2 (nt 175,964 to 199,240) was similar to that of plasmid pKP18-31-IMP, KPC (94% coverage, 99% identity). Resistance genes *msrE and mphE* were located on an IS*CR1*-IS*26*-mediated composite transposon, organized as *ISCR1-msrE-mphE-hp-repB-IS26*. In addition, the third variable region VR3 (nt 212,456 to 217,506, 5051 bp) contained the *tetD* resistance gene organized as *IS26-tetR-tetD-frmA-frmB-IS26*.

### 3.6. Genomic Analysis of Plasmid pB106-2

Belonging to IncFII incompatibility groups, the size of plasmid pB106-2 was 189,346 bp, with the average G+C content at 51.74% and the total number of predicted CDs at 228. The plasmid pB106-2 exhibited similarity (94% coverage, 99% identity) with an unnamed plasmid (unnamed1, accession number: CP033823.1) from the *Klebsiella* sp. FDAARGOS_511 strain. The plasmid pB106-2 contained two replicons, IncFII and IncFIB, but with none of the antimicrobial resistance gene.

### 3.7. Genomic Analysis of Plasmid pB106-IMP

The size of plasmid pB106-IMP was 85,326 bp, with the average G+C content at 48.80% and the total number of predicted CDs at 132. The backbone of plasmid pB106-IMP included sequences responsible for plasmid maintenance (*stbC* and *stbA* genes), antirestriction function (*adrA*, *adrR* and *adrK* genes), UV protection (*mucA* and *mucB* genes) and conjugative transfer (*traM*, *trbA* and *trbK* genes).

It is noteworthy that two different replication genes were found in the backbone regions of plasmid pB106-IMP, including the IncN-type replication gene *repE* and the IncX3-type replication gene *repB*. Remarkably, approximately 43.37% of the sequences were similar to a series of IncX3-type plasmids (identity > 99%, coverage 100%) and 51.92% of the sequences were similar to a series of IncN-type plasmids (identity 100%, coverage > 99%), with both regions accounting for more than 95% of the whole sequences of pB106-IMP. In addition, these two regions were linked by a composite transposon, and this composite transposon was bracketed by two identical IS*26*, with a ESBLs gene *bla*_SHV-12_ inserted. Another resistance gene *bla*_IMP-4_ was located on the IS-mediated transposition unit, organized as *IS26-*Δ*IntI1-bla*_IMP-4_*-ItrA-mobC-IS6100*. A 54-bp segment of the integrase gene was deleted and the IS*26* was located at the 5’-end of In823 integrons. A similar structure was reported in the *bla*_IMP-4_-carrying plasmid pIMP-HZ1 (the GenBank accession number: KU886034.1) originating from the *K. pneumoniae* strain in China [28,29]. The sequence of *bla*_IMP-4_ was 100% identical to those frequently detected in the broad host range of IncN-type plasmids [28]. The most common genetic environments of *bla*_IMP-4_ were complex class 1 integrons (In809 and In823). In809 is a genetic structure with *bla*_IMP-4_ as the core (*bla*_IMP-4_*-qacG2-aacA4-catB3-*Δ*qacE*) and IS*26* nearby. In823 was first reported in plasmid pIMP-HZ1 from the *K. pneumoniae* strain [28,29], in which *bla*_IMP-4_ was captured as a cassette by the class 1 integrons. A type II intron, *Kl.pn.I3*, was located downstream of *bla*_IMP-4_ and a *qnrS1* (quinolone resistance gene) region was located upstream of In823. However, in our study, in In823, the upstream of truncated *IntI1* was IS*26* and a genetic structure of *bla*_SHV-12_ (*IS26*-*bla*_SHV-12_*-ygbI-IS26*) was found at the same variable region in plasmid pB106-IMP [30]. As a consequence, we hypothesized that the formation of the variable region in plasmid pB106-IMP occurs due to the recombination event mediated by the transposition formed by two copies of IS*26* in the transposon.

## 4. Discussion

In this study, the whole genomic sequences of the *K. michiganensis* strain KMIB106 were analyzed, including a chromosome and three mega plasmids. For the profile of resistance genes, only two resistance genes, *oqxB* and *bla*_OXY-4-1_, were located on the chromosome. As previously found, bacteria carrying the resistance gene *bla*_OXY-4-1_ showed natural resistance to amino and carboxy penicillins. In comparison with the chromosome, plasmid pB106-1 and pB106-IMP were found to carry a large number of resistance genes which are associated with resistance to cephalosporins, β-Lactamase inhibitors, carbapenems, tetracycline antibiotics and compound sulfamethoxazole. Coincidentally, the *K. michiganensis* strain KMIB106 exhibited antimicrobial resistance to such antibiotics. Thus, the large proportion of the resistance genes that are likely relevant to the resistance phenotypes in KMIB106 were located on plasmids, which strongly suggested such mega plasmids with various resistance genes are the crucial factor responsible for the multidrug resistance of the host strain. Thus, horizontal gene transfer mediated by mobile genetic elements, such as plasmids, transposons and integrons, plays a major role in the dissemination of antibiotic resistance genes, as well as the ultimate adaptation of the host strain to the antibiotic pressure.

**Figure 2:**
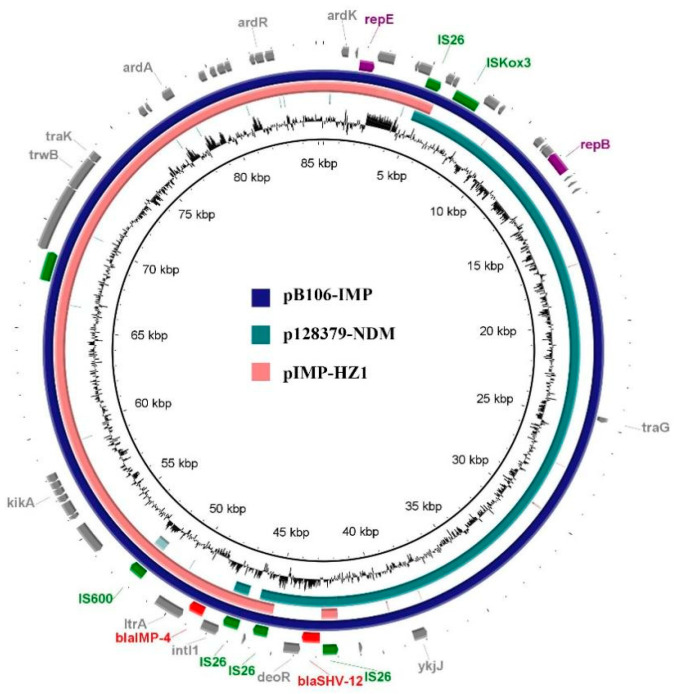
Whole sequence of pB106-IMP and comparison of p128379-NDM, pIMP-HZ1. Plasmid pB106-IMP was used as a reference to compare with the other two plasmids. The black cycle presents the GC content of pB106-IMP. The outer two rings comprise the coding sequences of pB106-IMP and key features are present in different colors: resistance genes are in red, replicon genes are in purple, other genes are in grey and mobile elements are in green.

**Figure 3:**
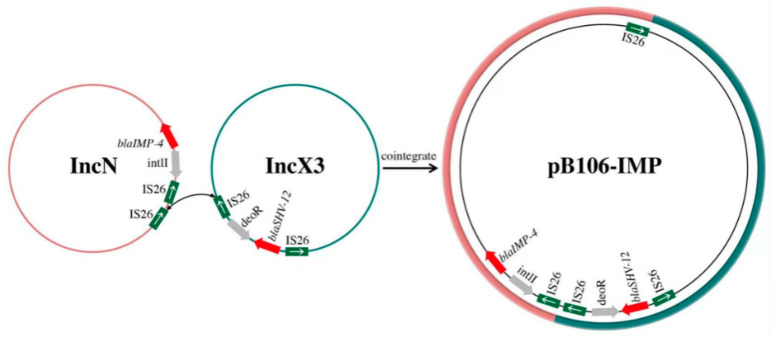
Proposed fusion mechanism of the cointegrate plasmid pB106-IMP mediated by IS*26*. IS*26*s are shown as green squares (the white arrow in green square indicates the orientation of IS*26*), resistance genes are shown as red arrows and other genes are shown as grey arrows, pink circle represents the IncN plasmid and pink circle represents the IncX3 plasmid.

In general, each plasmid has one specific replicon gene and a plasmid might be driven away by other plasmids if they carry an incompatible replicon. However, recently, a large number of mega plasmids containing two or even more functional replicons have been reported. Importantly, the carriage of multiple replicons in one single plasmid may allow these plasmids to contain more than one incompatible replicon, and thus expand the host range and further facilitate the dissemination of these plasmids. Concerning *bla*_IMP-4_ and *bla*_shv-12_, the plasmids containing *bla*_IMP-4_ often belong to several replicon types, including IncN, IncHI2, IncF, IncL/M and IncI1 [31,32,33], and the plasmid replicon types associated with the widespread dissemination of *bla*_shv-12_ include IncX3, IncA/C, IncF, IncHI2, IncI1α/γ, IncL/M, IncN, IncP, IncR, and IncK [34]. In our study, the plasmid pB106-IMP can be divided into three regions, including the IncN section, the IncX3 section and the 4029 bp segment carried *bla*_SHV-12_. The IncN section was similar to an IncN plasmid pIMP-HZ1, containing several conjugative transfer genes which implies the potential capability of horizontal transmission by conjugation for plasmid pB106-IMP. The IncX3 section of plasmid pB106-IMP was identical to an IncX3 plasmid p128379-NDM (the GenBank accession number: MF344560.1) which carries both *bla*_NDM-1_ and *bla*_SHV-12_ genes. In comparison, the 4029 bp *bla*_SHV-12_ segment in plasmid pB106-IMP linking the IncN and IncX3 section was likely derived from the plasmid p128379-NDM (100% coverage, 99.96% identity). Furthermore, there were three identical IS*26* fragments in plasmid pB106-IMP which were located at the junction of the IncX3 section; the 4029 bp segment carried *bla*_SHV-12_ and IncN section, respectively. As a consequence, it could be inferred that the cointegrate plasmid pB106-IMP was formed by fusion of an IncX3 plasmid and an IncN plasmid, with the IS*26* structure located between these two sections (Figure 2, Figure 3). Notably, this is the first report of an IncN and IncX3 cointegrate plasmid. As a novel IncN-IncX3 cointegrate plasmid, pB106-IMP is potentially capable of facilitating the dissemination of *bla*_shv-12_ and *bla*_IMP-4_ among *Klebsiella* spp.

ISs are simple autonomous mobile elements that mediate recombination events within genomes and further cause structural rearrangements. For a genetic structure composed of terminal inverted repeats and one ORF-encoded transposase, the terminal inverted repeats function as the specific recognition sites for the transposase, and the transposase further conducts the strand cleavage and transfer reactions. Various ISs are reported to play critical roles in the formation of these cointegrate plasmids [35]. IS*26*, as an important member of the IS*6* family, contains an 820 bp nucleotide sequence, with two inverted repeats and only one gene *tnp26* encoding 234 amino acids. IS*26* has been reported to play an important role in genetic mobility in plasmids and chromosomes among Enterobacteriaceae, which reorganize plasmids by replicative transposition or targeted conservative [36].

## 5. Conclusions

This study, for the first time, described a novel IncN-IncX3 cointegrate plasmid pB106-IMP. It contained two replicons and two carbapenem genes *bla*_IMP-4_ and *bla*_SHV-12_, giving its nature host KMIB106 carbapenem-resistance. Our work first expanded the diversity of the plasmids incompatibility group in *Enterobacterales.* Revealing the mechanisms underlying the formation of this novel cointegrate plasmid will help to find new ways to inhibit dissemination of resistance genes through this type of plasmid.

## Figures and Tables

**Table 1:** Antimicrobial susceptibility profiles for KMIB106.

Group	Antibiotics	Breakpoints (μg/mL)		MIC (μg/mL)
		S	R	
Fluoroquinolones	Ciprofloxacin	≤0.25	≥1	≤0.25
	Levofloxacin	≤0.5	≥2	≤0.12
Beta-lactams	Piperacillin/tazobactam	≤16/4	≥128/4	≥128/4
	Amoxicillin/clavulanic acid	≤8/4	≥32/16	≥32/16
	Ticacillin/clavulanic acid	≤16/2	≥128/2	≥128/2
	Ceftriaxone	≤1	≥4	≥64
	Ceftazidime	≤4	≥16	≥64
	Cefoxitin	≤8	≥32	≥64
	Cefuroxime axetil	≤8	≥32	≥64
	Cefuroxime sodium	≤8	≥32	≥64
	Aztreonam	≤4	≥16	≤1
	Cefepime	≤2	≥16	≥32
	Imipenem	≤1	≥4	≥16
	Ertapenem	≤0.5	≥2	≥8
Tetracyclines	Tigecycline	≤2	≥8	≤0.5
	Minocyline	≤4	≥16	≥16
	Doxycycline	≤4	≥16	≥16
Aminoglycosides	Amikacin	≤16	≥64	≤2
	Tobramycin	≤4	≥16	≤1
Folate pathway inhibitors	Compound sulfamethoxazole	≤2/38	≥4/76	≥320/80
Lipopeptides	Colistin	/	≥4	≤0.5

“/” indicates that the interpretive standard does not exist in Clinical and Laboratory Standards Institute. R, resistant; S, susceptible; MIC, minimum inhibitory concentration.

**Table 2:** Resistant determinants identified by genomic for KMIB106.

Resistance Gene	Location	Identity	Query/Template Length	Position	Predicted Phenotype	Accession Number
*oqxB*	chromosome	99.10	3131/3153	280,484..283,614	FluoroquinoloneResistance	EU370913
*bla_O_*_XY-4-1_	chromosome	99.88	869/873	5,016,109..5,016,977	Beta-lactam resistance	AY077481
*tetD*	pB106-1	100.0	1185/1185	213,924..215,108	TetracyclineResistance	AF467077
*mphA*	pB106-1	100.0	906/906	82,715..83,620	Macrolide resistance	D16251
*msrE*	pB106-1	100.0	1476/1476	188,852..190,327	Macrolide resistance	FR751518
*mphE*	pB106-1	100.0	885/885	190,383..191,267	Macrolide resistance	DQ839391
*ARR-3*	pB106-1	100.0	453/453	90,760..91,212	Rifamycin resistance	JF806499
*addA16*	pB106-1	99.65	846/846	89,128..89,973	Aminoglycosides Resistance	EU675686
*fosA3*	pB106-1	100.0	417/417	81,087..81,503	Fosfomycin resistance	AB522970
*sul1*	pB106-1	100.0	840/840	87,831..88,670	Folate pathway antagonist	U12338
*dfrA27*	pB106-1	100.0	474/474	90,154..90,627	Folate pathway antagonist	FJ459817
*bla*_SHV-12_	pB106-IMP	98.88	861/861	42,832..43,692	Beta-lactam resistance	KF976405
*bla*_IMP-4_	pB106-IMP	100.0	741/741	48,488..49,228	Beta-lactam resistance	AF244145

## Data Availability

Not applicable.

## Supplementary Materials

The following supporting information can be downloaded at: https://www.mdpi.com/article/10.3390/antibiotics12091463/s1, Figure S1: Sequencing depth distribution map.
