# Supplementary figures and images for: Resistome and Genome Analysis of an Extensively Drug-Resistant Klebsiella michiganensis KMIB106: Characterization of a Novel KPC Plasmid pB106-1 and a Novel Cointegrate Plasmid pB106-IMP Harboring blaIMP-4 and blaSHV-12
Source: Antibiotics (Basel). 2023 Sep 20;12(9):1463. doi: 10.3390/antibiotics12091463 (PMC10525660; doi:10.3390/antibiotics12091463)

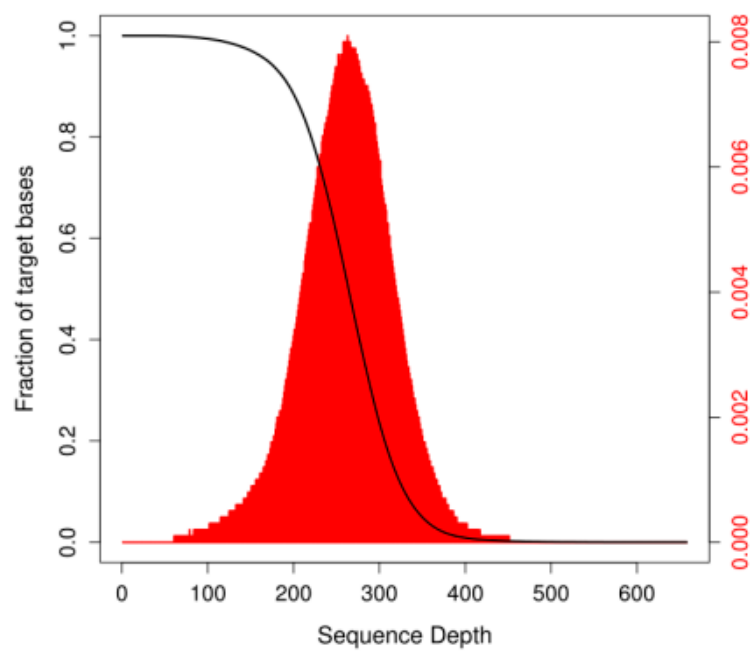

Figure S1. Sequencing depth distribution map.

Supplement: Supplementary file 1 [file antibiotics-12-01463-s001.zip › antibiotics-2531863-supplementary.pdf]
